# Long‐Term Follow‐up After Percutaneous Coronary Interventions for Chronic Total Occlusions

**DOI:** 10.1002/clc.70414

**Published:** 2026-07-16

**Authors:** Rémi Arnold, Richard Gervasoni, Bertrand Ledermann, Lionel Moulis, Sonia Soltani, Benoit Lattuca, Pierre Robert, Florence Leclercq

**Affiliations:** ^1^ Cardiology Department University Hospital of Montpellier Montpellier France; ^2^ Cardiology Department University Hospital of Nîmes Nîmes France; ^3^ Clinical Research and Epidemiology Unit University Hospital of Montpellier Montpellier France

**Keywords:** chronic total occlusions, LVEF, major cardiovascular events, NYHA, percutaneous coronary intervention, survival

## Abstract

**Background:**

Percutaneous coronary interventions (PCI) for chronic total occlusions (CTO) remain challenging procedures with controversial long‐term clinical benefits.

**Methods:**

We conducted an observational monocentric study including consecutive patients who underwent PCI for CTO by experienced operators in a French University Hospital between January 2015 and December 2022. All patients had symptoms and/or proven myocardial ischemia. Patients were divided into two groups based on PCI success or failure. The primary endpoint was the occurrence of MACE, defined as the composite of cardiac death, non‐fatal myocardial infarction, and target vessel revascularization up to 8 years of follow‐up.

**Results:**

Of the 448 patients who underwent a CTO‐PCI, 401 (89.5%) had a successful procedure, while 47 (10.5%) experienced a failed intervention. During a mean follow‐up of 3.7 years, MACE occurred in 71 patients (15.8%), including 12 patients (25.5%) in the failed group and 59 patients (14.7%) in the successful group (HR 2.68 [1.42; 5.06]; *p* < 0.01). While both groups had similar baseline clinical risk profiles, the failed CTO‐PCI group had a lower overall survival rate (74.5% vs. 88.8%; HR 2.23 [1.17; 4.27]; *p* = 0.02). The successful CTO‐PCI group showed significant improvements in left ventricular ejection fraction (LVEF) (*p* < 0.01) and NYHA class (*p* < 0.01). The J‐CTO score was the only predictor of procedural failure (1.96 [1.44; 2.67]; *p* < 0.01).

**Conclusions:**

Failed CTO‐PCI was associated with increased incidence of MACE. LVEF and NYHA class was improved in successful CTO‐PCI, supporting the pursuit of this procedure in selected cases.

## Summary

- CTO recanalization was achieved in 90% of patients.
- Successful CTO‐PCI was associated with a significant reduction in MACE.
- Successful CTO‐PCI improved LVEF and NYHA functional class.

## Graphical abstract

In a single‐center cohort of 448 CTO patients, successful PCI (89.5%) was associated with significantly lower MACE, improved survival, better LVEF, reduced ischemia, and fewer cardiac rehospitalizations compared with failed procedures.

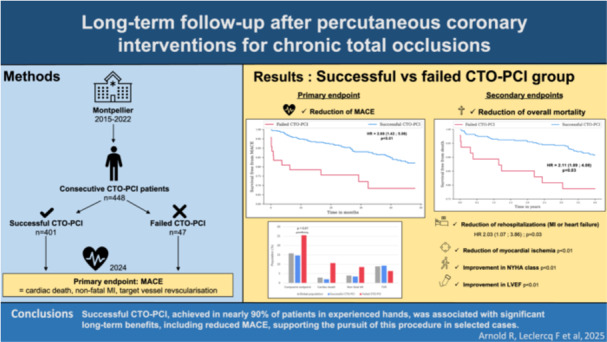

## 1 Introduction

Chronic total occlusions (CTOs) are commonly encountered, appearing in 15%−25% of coronary angiograms [1, 2]. However, percutaneous coronary intervention (PCI) for coronary CTO is considered to be one of the most challenging procedures in interventional cardiology.

Despite evidence from previous randomized and observational studies [3, 4, 5, 6] suggesting potential clinical benefits, only 10% of CTOs are treated with PCI in clinical practice [7]. This low angioplasty rate, compared with coronary artery bypass grafting (CABG) and medical therapy alone, may be explained by the anticipated complexity of the procedure, which requires specialized equipment [8, 9] and techniques [10, 11], experienced operators, higher radiation exposure, and contrast volume. There are also concerns regarding complications such as coronary perforation and cardiac tamponade. Only three randomized clinical trials [12, 13, 14] have compared PCI with optimal medical therapy (OMT) in terms of symptoms and mortality, and their findings remain controversial [15].

Whether PCI for CTO lesions improves long‐term clinical outcome remains unclear. In this contemporary observational study, we therefore aimed to describe clinical outcomes in a retrospective cohort of patients who underwent PCI and to evaluate the impact of successful CTO‐PCI on long‐term major adverse cardiovascular events (MACE), survival, rehospitalizations, symptoms, left ventricular ejection fraction (LVEF), and myocardial ischemia up to 8‐year follow‐up.

## 2 Methods

### 2.1 Study Design

All consecutive patients in whom percutaneous recanalization of a CTO was attempted at Montpellier University Hospital (France) between January 2015 and December 2022 were screened for eligibility. Only patients with symptoms and/or reversible myocardial ischemia in the territory of the occluded artery and myocardial viability were included in this study.

Symptoms were defined as angina pectoris greater than Canadian Cardiovascular Society (CCS) class II [16] or dyspnea exceeding New York Heart Association (NYHA) functional class II [17].

Reversible myocardial ischemia was defined as (1) an ischemic area involving ≥ 10% of the LV myocardium using stress single‐photon emission computed tomography (SPECT), or (2) stress‐induced hypokinesia or akinesia affecting ≥ 3 of 16 LV segments on stress echocardiography (TTE) [18].

Myocardial viability was assessed by cardiac MRI (magnetic resonance imaging), defined as transmural segmental late gadolinium enhancement < 25% in the affected segment, dobutamine stress TTE demonstrating segmental contractile reserve, or by resting TTE confirming the absence of segmental akinesia or left ventricular wall thinning in the CTO territory prior to inclusion [19].

This study protocol was approved by an independent ethics committee (CSE, Comité Scientifique et Ethique du CHU de Montpellier, approval number 2024‐01‐008). The study was registered on clinicaltrials.gov (NCT06544174).

### 2.2 Definitions

A coronary CTO was defined as a thrombolysis in myocardial infarction (TIMI) grade 0 flow within an occluded coronary artery segment > 2.5 mm in diameter, with an estimated occlusion duration > 3 months [20].

The probability of successful anterograde guidewire crossing was assessed using the Japanese CTO score (J‐CTO) [21], which ranges from 0 to 5.

Procedural success was defined as achieving a final residual stenosis < 30% by visual estimation and a TIMI grade 3 flow after CTO recanalization [22] in all living patients within 24 h following angioplasty.

Baseline characteristics were collected including cardiovascular risk factors, body mass index (BMI), baseline LVEF, history of prior MI, CABG or angioplasty, and medical therapy at hospital discharge.

### 2.3 CTO Procedure

All CTO procedures were performed by two experienced operators.

Periprocedural antithrombotic management followed contemporary standards. In patients not previously receiving a P2Y12 inhibitor, a 600 mg loading dose of clopidogrel was administered before the intervention. For those already treated with an alternative P2Y12 inhibitor, the ongoing therapy was maintained without additional loading. At the start of the procedure, intravenous aspirin (250 mg) and unfractionated heparin (100 IU/kg) were administered. Anticoagulation was adjusted to maintain an activated clotting time (ACT) between 250 and 300 s, with repeated ACT measurements approximately every 30 min and supplemental heparin given as needed. Following discharge, patients were prescribed dual antiplatelet therapy (DAPT) combining aspirin (75 or 100 mg daily) and clopidogrel (75 mg daily) for 12 months. In patients requiring anticoagulation, triple antithrombotic therapy was limited to a maximum duration of 1 month in accordance with current guideline recommendations [23].

Anterograde or retrograde techniques and equipment (guidewires and microcatheters) were employed in line with recent recommendations [24].

### 2.4 Study Endpoints

The primary endpoint was the occurrence of a MACE, defined as a composite of cardiac death, non‐fatal myocardial infarction (MI), and new target vessel revascularization (TVR). Time‐to‐event was calculated from the index procedure to the occurrence of the event, death, or study endpoint.

Cardiac death was defined as death resulting from MI, heart failure, or sudden cardiac death.

Non‐fatal MI was defined according to the third universal definition of MI [25].

TVR referred to any percutaneous or surgical revascularization of the treated CTO artery.

Secondary endpoints included the individual components of the composite endpoint and in‐hospital events.

In‐hospital procedural complications included death, periprocedural MI (type 4a), coronary perforation, pericardial tamponade requiring drainage, local vascular complications, major bleeding, contrast‐induced nephropathy, and stroke.

Periprocedural MI (type 4a) was diagnosed in the presence of an elevation of creatine kinase (CK) exceeding three times the normal upper limit [1, 6].

Coronary perforations were defined according to the Ellis classification [26].

Contrast‐induced nephropathy was characterized by a 24 h post‐procedural increase in creatinine of more than 25% from baseline [27].

Major bleeding was defined with a BARC classification [28] grade ≥ 3.

Secondary endpoints evaluated at the longest follow‐up also included:

− Cardiac‐related rehospitalizations, including congestive heart failure or MI.
− Symptom improvement.
− Restenosis is defined as a greater than 50% diameter stenosis at follow‐up angiography in the initially dilated total occlusion. Reocclusion was defined as the recurrence of total occlusion in the initially dilated total occlusion.
− Improvement in LVEF as measured by TTE at baseline and during follow‐up.
− Decrease in ischemic burden of ≥ 5% of the LV myocardium area in SPECT imaging, when this test was performed before and after CTO‐PCI.
− Identification of predictor factors of CTO procedure failure.

### 2.5 Follow‐up

All medical records were reviewed. Subsequent hospitalizations, coronary angiograms, and repeat revascularizations (PCI or CABG) at the Montpellier University Hospital were also collected from the clinical database. Follow‐up continued until August 31, 2024.

When information was incomplete, phone calls were made to patients to ask them about symptoms, hospitalizations following the index CTO angioplasty, and their most recent coronary angiogram. If patients could not be reached, information was collected from the referring cardiologist.

All deaths were recorded and classified as either cardiac or non‐cardiac when the information was available.

### 2.6 Statistical Analyses

#### 2.6.1 Sample Size

In a previous observational study, the occurrence of MACE at Year 4 was described as 15% in the group with CTO success and 43% in the group with CTO failure [29]. With these numbers and estimating that in our population there would be about 10 times more success than failure and the censoring rate would be 0.2, a number of 251 patients (226 in the success group and 25 in the failure group) was needed to have a power higher than 90% to detect a difference between the two survival curves in a log rank test with a two‐sided 5% *⍺* [30, 31].

#### 2.6.2 Statistical Plan

Numeric variables were expressed as mean (±SD) and discrete outcomes as absolute and relative (%) frequencies. Follow‐up duration was also expressed as median (Q25; Q75). Patients were divided into two groups according to angiographic success or failure. Group comparability was assessed by comparing baseline demographic data and follow‐up duration between groups. Normality and heteroskedasticity of continuous data were assessed with the Shapiro−Wilk and Levene's test, respectively. Continuous outcomes were compared with the unpaired Student *t*‐test, Welch *t*‐test, or Mann−Whitney *U* test according to data distribution. Discrete outcomes were compared with chi‐squared or Fisher's exact test, accordingly.

To compare time‐to‐event variables between the two groups, we used the Kaplan−Meier method to estimate survival probabilities and their 95% confidence intervals, and used the log‐rank test. To evaluate factors associated with the occurrence of MACE or overall death, we used a multivariate Cox regression after checking its conditions of application and the non‐multicollinearity of the explanatory variables, which were selected based on existing literature: age, gender, diabetes, LVEF, prior MI, prior PCI, prior CABG, renal disease, peripheral arterial disease, and SYNTAX score [6].

The *⍺* risk was set to 5% and two‐tailed tests were used. Statistical analysis was performed with EasyMedStat (version 3.36; www.easymedstat.com).

## 3 Results

### 3.1 Study Population

**Figure 1:**
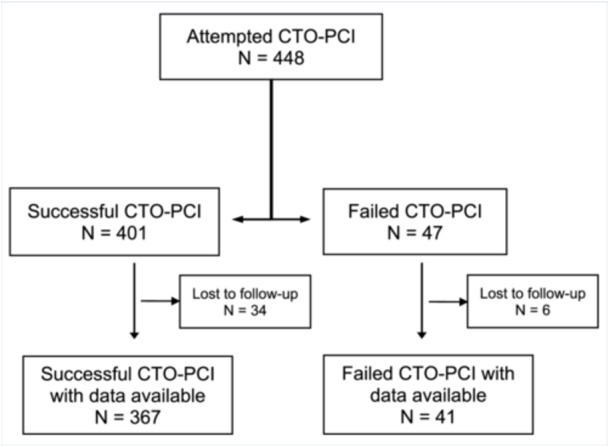
Flowchart.

A total of 448 patients underwent CTO‐PCI between January 2015 and December 2022. Procedural success was achieved in 401 patients (89.5%), whereas 47 patients (10.5%) experienced procedural failure (Figure 1). Mean follow‐up duration was 3.7 years (± 2.4 years), with a median of 3.5 years (interquartile range: 2.0–5.5 years) and a maximum follow‐up of 8 years.

Baseline characteristics were well balanced between groups (Table 1). Medical therapy at discharge was similarly optimized in both groups, with no statistically significant differences observed between them.

**Table 1:** Baseline patients' characteristics.

Variable	Overall population	Successful CTO‐PCI	Failed CTO‐PCI	
		*N* = 401	*N* = 47	*p* value
Gender				0.490
Men	390 (87.1%)	347 (86.53%)	43 (91.49%)	
Women	58 (12.9%)	54 (13.47%)	4 (8.51%)	
Age (years)	64.66 (±10.45)	64.38 (±10.38)	67.02 (±10.94)	0.061
BMI (kg/m^2^)	27.37 (±4.83)	27.4 (±4.84)	27.12 (±4.78)	0.970
Hypertension	267 (59.6%)	236 (58.85%)	31 (65.96%)	0.434
Diabetes	142 (31.7%)	122 (30.42%)	20 (42.55%)	0.127
Hypercholesterolemia	250 (55.8%)	218 (54.36%)	32 (68.09%)	0.102
History of smoke	296 (66.1%)	269 (67.08%)	27 (57.45%)	0.247
Family history of CAD	75 (16.7%)	66 (16.46%)	9 (19.15%)	0.679
Prior PCI	287 (64.1%)	256 (63.84%)	31 (65.96%)	0.900
Prior CABG	42 (9.4%)	36 (8.98%)	6 (12.77%)	0.424
Prior MI	218 (48.7%)	194 (48.38%)	24 (51.06%)	0.846
Peripheral vascular disease	53 (11.8%)	47 (11.72)	6 (12.77)	0.812
Renal dysfunction^a^	42 (9.4%)	37 (9.22%)	5 (10.64%)	0.79
LVEF (%)	50.57 (±11.60)	50.68 (±11.31)	49.61 (±14.03)	0.973
Clinical presentation				0.353
Stable angina	170 (37.95%)	155 (38.65%)	15 (31.91%)	
Dyspnea or congestive heart failure	126 (28.12%)	109 (27.18%)	17 (36.17%)	
Silent ischemia	78 (17.41%)	68 (16.96%)	10 (21.28%)	
Acute coronary syndrome	74 (16.52%)	69 (17.21%)	5 (10.64%)	
CCS classification				
Class 1	266 (59.38%)	235 (58.6%)	31 (65.96%)	0.052
Class 2	61 (13.62%)	59 (14.71%)	2 (4.26%)	
Class 3	79 (17.63%)	73 (18.2%)	6 (12.77%)	
Class 4	42 (9.37%)	34 (8.48%)	8 (17.02%)	
NYHA classification				
Class 1	300 (66.96%)	273 (68.08%)	27 (57.45%)	0.376
Class 2	71 (15.85%)	60 (14.96%)	11 (23.4%)	
Class 3	36 (8.04%)	32 (7.98%)	4 (8.51%)	
Class 4	41 (9.15%)	36 (8.98%)	5 (10.64%)	
Medication				
Aspirin	438 (97.77%)	392 (97.75%)	46 (97.87%)	> 0.999
Betablockers	356 (79.46%)	321 (80.05%)	35 (74.47%)	0.581
ACE‐I or ARBs	332 (74.11%)	301 (75.06%)	31 (65.95%)	0.241
MRA	68 (15.18%)	62 (15.46%)	6 (12.77%)	0.83
Vasodilators	128 (28.57%)	110 (27.43%)	18 (38.3%)	0.165
Statin	436 (97.32%)	390 (97.26%)	46 (97.87%)	> 0.999

*Note:* Data are expressed as mean ± SD, or *n* (%) of patients.

Abbreviations: ACE‐i, angiotensin‐converting enzyme inhibitors; ARBs, angiotensin receptor blockers; BMI, body mass index; CABG, coronary artery bypass grafting; CAD, coronary artery disease; CCS, Canadian Cardiovascular Society; LVEF, left ventricular ejection fraction; MI, myocardial infarction; MRA, mineralocorticoid receptor antagonist; NYHA, New York Heart Association; PCI, percutaneous coronary intervention.

a Renal dysfunction was defined as an estimated glomerular filtration rate < 60 mL/min/1.73 m^2^ of the body surface area.

### 3.2 Angiographic and Procedural Characteristics

Angiographic and procedural characteristics are reported in Table 2.

**Table 2:** Angiographic and procedural characteristics.

Variable	Overall population	Successful CTO‐PCI (*N* = 401)	Failed CTO‐PCI (*N* = 47)	*p* value
Target CTO vessel				
LAD	143 (31.9%)	128 (31.92%)	15 (31.91%)	> 0.999
LCA	110 (24.6%)	97 (24.19%)	13 (27.66%)	0.731
RCA	230 (51.3%)	207 (51.62%)	23 (48.94%)	0.846
Multiple CTOs (> 1)	28 (6.3%)	24 (5.99%)	4 (8.51%)	0.510
Mean J‐CTO score	1.75 (±1.19)	1.64 (±1.15)	2.72 (±1.12)	**< 0.001**
J‐CTO score				**< 0.001**
0 point	54 (14.9%)	53 (16.41%)	1 (2.56%)	
1 point	108 (29.8%)	104 (32.2%)	4 (10.26%)	
2 points	110 (30.4%)	98 (30.34%)	12 (30.77%)	
3 points	59 (16.3%)	48 (14.86%)	11 (28.21%)	
4 points	26 (7.2%)	16 (4.95%)	10 (25.64%)	
5 points	5 (1.4%)	4 (1.24%)	1 (2.56%)	
Number of diseased vessels				0.391
Single vessel: CTO lesion	120 (26.8%)	110 (27.43%)	10 (21.28%)	
Two vessels	202 (45.1%)	182 (45.39%)	20 (42.55%)	
Three vessels	126 (28.1%)	109 (27.18%)	17 (36.17%)	
SYNTAX score	16.91 (±5.88)	17.58 (±6.63)	18.46 (±6.9)	0.692
Vascular access				0.601
Radial approach	173 (38.6%)	157 (39.15%)	16 (34.04%)	
Femoral approach	275 (61.4%)	244 (60.85%)	31 (65.96%)	
Technic				**0.018**
Anterograde approach	414 (92.4%)	375 (93.52%)	39 (82.98%)	
Retrograde approach	34 (7.6%)	26 (6.48%)	8 (17.02%)	
Use of specific material				**0.008**
Rotablator atherectomy	11 (2.5%)	6 (1.5%)	5 (10.64%)	
Laser	4 (0.9%)	4 (1.0%)	0 (0.0%)	
Peak CK (UI/L)	153.36 (±218.47)	137.15 (±149.6)	292.73 (±500.12)	**0.006**
Contrast volume (mL)	193.55 (±93.17)	187.91 (±90.63)	246.26 (±102.29)	**< 0.001**
Radiation dose (cGy.m^2^)	9940.66 (±8546.24)	9503.2 (±8500.56)	14 435.9 (±7971.88)	**< 0.001**
CTO‐PCI attempt				
First attempt	397 (88.6%)	361 (90.02%)	36 (76.6%)	**0.006**
Second attempt	43 (9.6%)	35 (8.73%)	8 (17.02%)	
Third attempt	8 (1.8%)	5 (1.25%)	3 (6.68%)	

*Note:* Data are expressed as mean ± SD, or *n* (%) of patients. Bold values are statistically significant.

Abbreviations: CK, creatine kinase; CTO, coronary total occlusion; J‐CTO score, Japanese CTO score; LAD, left anterior descending; LCA, left circumflex artery; RCA, right coronary artery.

The overall success rate was 80.6% after the first attempt, increasing to 88.4% after a second attempt and 89.5% after a third attempt.

The mean J‐CTO score was significantly higher in the failed CTO‐PCI group compared with the successful CTO‐PCI group (2.72 ± 1.12 vs. 1.64 ± 1.15, *p* < 0.001).

Groups were similar regarding the number of diseased vessels and SYNTAX score (*p* = 0.39 and *p* = 0.69, respectively).

### 3.3 Complications and Procedure Safety

Intra‐hospital events and complications are presented in Table 3.

**Table 3:** Intra‐hospital events and complications.

Variable	Overall population	Successful CTO‐PCI (*N* = 401)	Failed CTO‐PCI (*N* = 47)	*p* value
Periprocedural death	2 (0.4%)	0 (0.0%)	2 (4.26%)	**0.011**
Local vascular complication				> 0.999
Hematoma	8 (1.8%)	8 (2.0%)	0 (0.0%)	
False aneurysm	8 (1.8%)	8 (2.0%)	0 (0.0%)	
Arteriovenous fistula	1 (0.2%)	1 (0.25%)	0 (0.0%)	
Stroke	2 (0.4%)	1 (0.25%)	1 (2.13%)	0.199
Contrast‐induced nephropathy	9 (2.0%)	7 (1.75%)	2 (4.26%)	0.242
Periprocedural MI	14 (3.1%)	8 (2.0%)	6 (12.77%)	**0.002**
Major bleeding (BARC ≥ 3)	8 (1.8%)	6 (1.5%)	2 (4.26%)	0.201
Coronary perforation	15 (3.3%)	11 (2.74%)	4 (8.51%)	0.061
Cardiac tamponade	8 (1.8%)	6 (1.5%)	2 (4.26%)	0.201

*Note:* Data are expressed as *n* (%) of patients. Bold values are statistically significant.

Abbreviation: MI, myocardial infarction

In‐hospital complications included two periprocedural deaths, both in patients with severely impaired LVEF (35%) at baseline. Coronary perforation occurred in 15 patients (11 in the successful and four in the failed group, *p* = 0.061), with eight requiring drainage (*p* = 0.201). Periprocedural MI was more common in the failed group (12.77% vs. 2.0%, *p* = 0.002).

### 3.4 Primary Outcome

**Figure 2:**
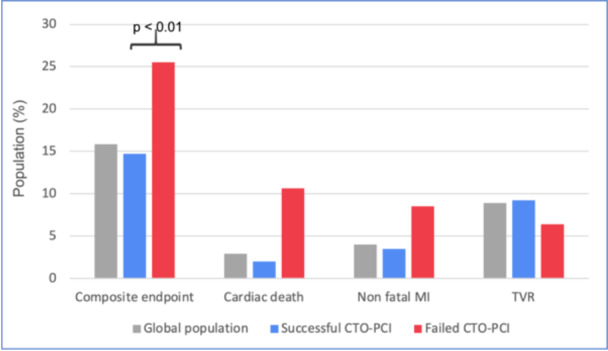
Composite endpoint and its components in the overall population and the two groups.

**Figure 3:**
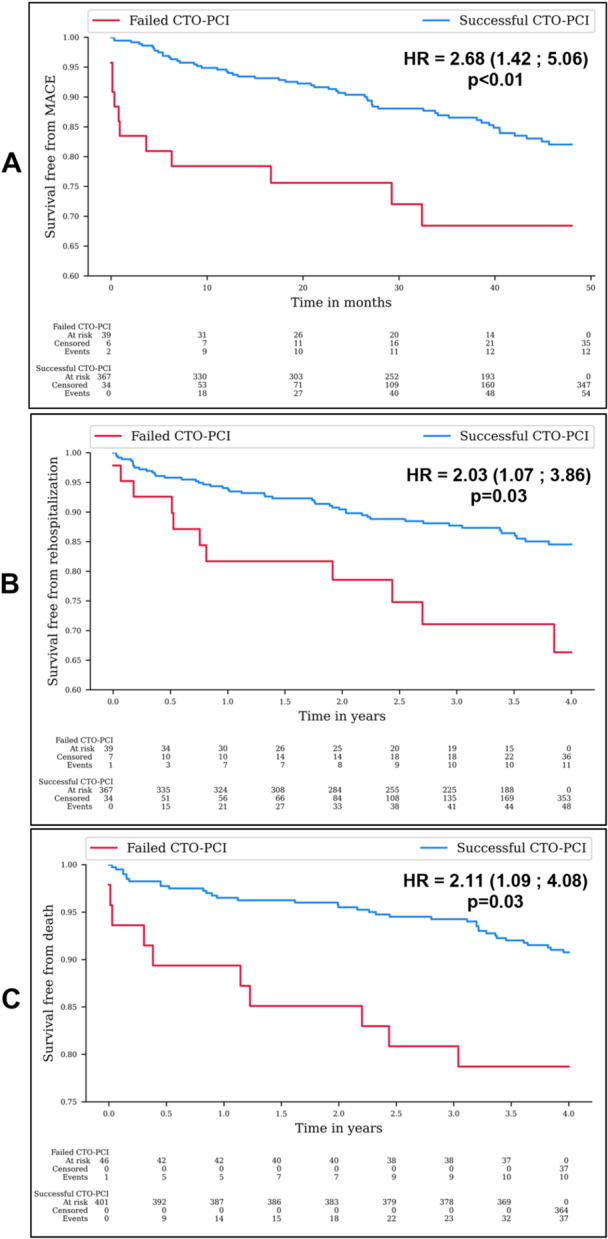
Time to event curves for MACE, rehospitalization, and all‐cause death. Kaplan−Meier curves for free from (A) MACE, (B) rehospitalization for cardiac cause, and (C) all‐cause death according to the success of the CTO recanalization.

The primary outcome occurred in 71 patients (15.8%), including 59 out of 401 patients (14.7%) in the successful CTO‐PCI group and 12 out of 47 patients (25.5%) in the failed CTO‐PCI group (*p* = 0.002) (Table 4). At 4 years, the cumulative incidence of MACE was 15.8%, with 14.7% in the successful group and 25.5% in the failed group (*p* = 0.002). The primary MACE driver was TVR in the successful group, while cardiac death predominated in the failed group (Figures 2 and 3).

**Table 4:** Clinical outcomes in the successful and failed CTO‐PCI groups.

Variable	Successful CTO‐PCI (*N* = 401)	Failed CTO‐PCI (*N* = 47)	Univariate hazard ratio (95% CI)	*p* value	Multivariate hazard ratio (95% CI)^a^	*p* value
Composite endpoint						
Cardiac death, non‐fatal MI, or TVR	59 (14.71%)	12 (25.53%)	**2.36 (1.27; 4.4)**	**0.007**	**2.68 (1.42; 5.06)**	**0.002**
All‐cause death	45 (11.22%)	12 (25.53%)	**2.63 (1.39; 4.97)**	**0.003**	**2.11 (1.09; 4.08)**	**0.027**
Cardiac death	9 (2.24%)	5 (10.64%)				
Non‐cardiac death	13 (3.24%)	3 (6.38%)				
Death from unknown cause	23 (5.74%)	4 (8.51%)				
Non‐fatal myocardial infarction	22 (5.49%)	4 (8.51%)	1.94 (0.669; 5.65)	0.222		
Target vessel revascularization	37 (9.23%)	6 (12.77%)	1.84 (0.776; 4.37)	0.166		
PCI	36 (8.98%)	3 (6.38%)				
CABG	1 (0.25%)	3 (6.38%)				
Other vessel revascularization	44 (10.97%)	5 (10.64%)	1.24 (0.491; 3.12)	0.651		
Rehospitalization	60 (14.96%)	11 (23.4%)	**2.03 (1.07; 3.86)**	**0.031**		
Congestive heart failure	38 (9.48%)	7 (14.89%)				
Myocardial infarction	22 (5.49%)	4 (8.51%)				

*Note:* Data are expressed as the number of events (% of population). Bold values are statistically significant.

Abbreviations: CABG, coronary artery bypass grafting; MACE, major cardiovascular events; PCI, percutaneous intervention.

a With adjustment for age, gender, diabetes, LVEF, prior MI, prior PCI, prior CABG, RD, PAD, and SYNTAX score.

### 3.5 All‐Cause Death

At 4 years, the survival rate was 89% in the successful CTO‐PCI group and 78% in the failed CTO‐PCI group (*p* = 0.005). Patients with failed CTO recanalization had a higher risk of death even after adjusting for covariates (multivariate HR 2.11 [1.09; 4.08], *p* = 0.0265) (Table 4) (Figure 3).

### 3.6 Rehospitalizations

Cardiac‐related rehospitalizations occurred in 15.8% of the overall population, 15% of patients in the successful CTO‐PCI group, and 23.4% in the failed CTO‐PCI group (Table 4). The risk of rehospitalizations was twice as high in the failed CTO‐PCI group (univariate HR 2.03 [1.07; 3.86], *p* = 0.03) (Figure 3).

### 3.7 Restenosis

One hundred and fourteen patients of the total population had a coronary angiogram during follow‐up (25% of the population). Of these, restenosis was observed in 22% of cases, and total reocclusion occurred in 13% of patients.

Restenosis was more frequent after retrograde approach compared with anterograde approach (75% and 32%, respectively, *p* = 0.02).

### 3.8 Predictors of Unsuccessful CTO Recanalization

**Figure 4:**
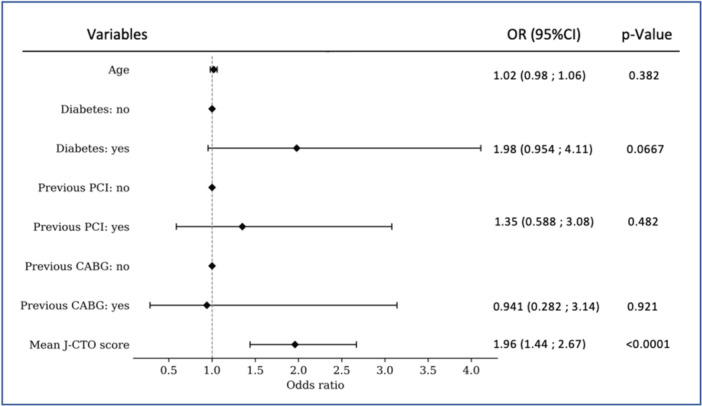
Multivariate predictors of unsuccessful CTO recanalization.

The mean J‐CTO score was the only independent predictor of CTO‐PCI failing (Figure 4). Factors such as diabetes, prior CABG, prior PCI, and age were not significantly associated with an increased risk of failed CTO recanalization.

### 3.9 Symptoms

**Figure 5:**
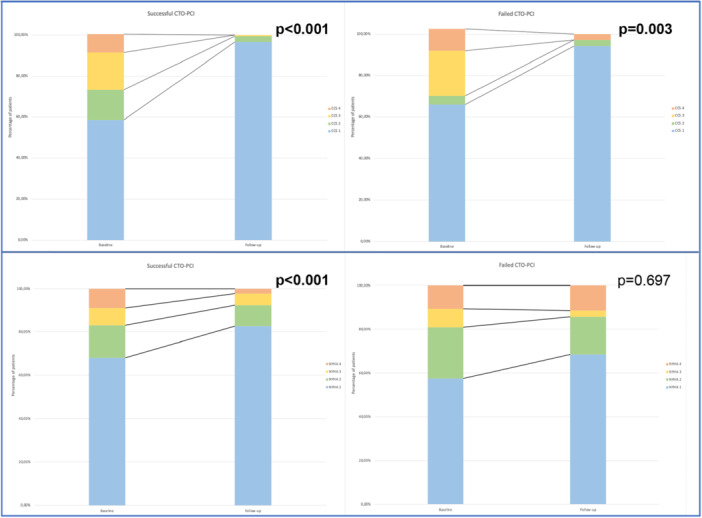
Comparison of changes in CCS classification and NYHA classification from baseline to follow‐up between successful and failed CTO‐PCI.

Angina pectoris improved significantly in both the successful (*p* < 0.001) and failed CTO‐PCI groups (*p* = 0.003) (Figure 5). However, the NYHA class improved only in the successful CTO‐PCI group (*p* < 0.001) and showed no improvement in the failed CTO‐PCI group (*p* = 0.697).

### 3.10 Improvement in LVEF

The median difference in LVEF as measured by TTE before and after the CTO procedure was significant only in the successful CTO‐PCI group. In this group, the median difference in LVEF was 5% (*p* < 0.01).

### 3.11 Reduction in Myocardial Ischemia

During follow‐up, 108 patients who had documented reversible myocardial ischemia prior to the CTO procedure underwent another ischemia test.

In the successful CTO‐PCI group, 72 patients showed reversible myocardial ischemia in the CTO myocardial territory, while 25 patients did not. In contrast, in the failed CTO‐PCI group, only two patients had documented reversible myocardial ischemia, and nine did not. The difference between groups was significant (*p* = 0.002). Patients with ischemia reduction had higher follow‐up LVEF (56.2 ± 7.1% vs. 50.0 ± 11.9%, *p* = 0.002).

## 4 Discussion

The present study aimed to describe the long‐term outcomes of patients undergoing PCI for CTO and to compare the prognosis of successful versus failed procedures in a contemporary cohort with follow‐up extending up to 8 years.

Key findings revealed that failed CTO‐PCI, despite OMT, was associated with a significantly higher incidence of MACE, mortality, and rehospitalization. In contrast, successful CTO recanalization was associated with improvement in NYHA functional class and LVEF, and a reduction in myocardial ischemia. The J‐CTO score emerged as the only independent predictor of procedural failure.

### 4.1 Patients and Procedural Characteristics

Previous observational and randomized studies have characterized CTO patients as typically older than 60 years, predominantly male, with a history of MI, preserved LVEF, and around 30% having diabetes. Our patient population aligns with these characteristics [6, 15].

Baseline characteristics were well balanced between the successful and failed groups, including multivessel disease burden, mean SYNTAX score, and use of OMT at discharge. However, we lacked comprehensive longitudinal data regarding medication adherence and adjustments after PCI, which may have influenced long‐term outcomes and should be considered when interpreting our findings.

CTO lesions were localized in RCA in half of patients, also consistent with previous studies [15]. The mean J‐CTO score was comparable to those reported in randomized studies, making our findings relevant to similar complex procedures [13, 14]. Regarding technical aspects, the retrograde approach was used in only 8% of procedures, compared with 24%–35% in recent studies [6, 13, 14]. However, the overall success rate was high (89% after multiple attempts), aligning with highest rates in current literature [32].

### 4.2 Primary Endpoint: Reduction of MACE

The overall incidence of MACE was 15.8%, with TVR being the primary driver, which aligns with findings from previous studies [6, 14]. This finding suggests that incomplete or suboptimal revascularization may lead to persistent ischemia or symptom recurrence, requiring additional intervention. These data emphasize the potential benefit of referring patients to high‐volume CTO centers to improve long‐term outcomes by maximizing procedural success rates.

Also, in a recent study including stable CAD [33], MACE occurred in 13.6% of this population, suggesting that outcomes in our CTO population are similar to those in stable coronary disease.

While most patients in both groups received OMT, and clinical and angiographic characteristics were similar (except regarding the J‐CTO score), MACE occurred twice as frequently in the failed CTO recanalization group, aligning with findings of a recent large‐scale meta‐analysis [34]. Although baseline discharge medical therapy did not differ significantly between groups, the absence of detailed longitudinal medication data prevents full adjustment for potential treatment‐related confounding. These results also suggest that the procedure itself could have impacted patient prognosis. The sustained divergence of event curves over follow‐up further supports the durability of the benefit associated with successful recanalization.

### 4.3 Clinical Endpoints

#### 4.3.1 All Cause‐Mortality

In this study, all‐cause mortality was statistically higher in the failed CTO‐PCI group. While randomized trials have not shown definitive prognostic benefit for CTO PCI [14, 35], observational data [29, 36] continue to suggest potential symptomatic and survival advantages in selected populations. Our study seeks to contribute real‐world insights. It is important to acknowledge that highly symptomatic or high‐risk subgroups—such as patients with proximal LAD CTO—are often underrepresented in randomized trials. Retrospective data suggest these patients may derive substantial benefit from successful recanalization. Underrepresentation of such subgroups in RCTs may attenuate the observed treatment effect and underestimate the benefit in clinical practice.

### 4.4 Restenosis

The observed 35% restenosis rate in our study is lower than that reported in older studies, conducted before the use of new‐generation drug‐eluting stents [4, 37].

The predominant use of the antegrade true‐lumen technique in our study may have contributed to this finding, as retrograde and dissection‐reentry strategies have been associated with higher restenosis rates [38].

However, the true restenosis rate may be underestimated as only 25% of patients had a coronary angiogram during follow‐up, typically in symptomatic patients rather than as routine control.

### 4.5 Symptoms

The CCS classification, which assesses angina symptoms, increased regardless of whether the CTO‐PCI was successful. Indeed, 70% of patients in the failed‐CTO group were asymptomatic at follow‐up. This improvement may reflect optimization of medical therapy in both groups. Additionally, baseline CCS class was assessed prior to any revascularization; therefore, patients with multivessel disease may have benefited from treatment of non‐CTO lesions, potentially explaining symptom relief even after failed CTO recanalization. In randomized DECISION‐CTO trial [14], no significant difference in symptoms was observed between CTO‐PCI group and OMT group, as 20% of patients the OMT group received non‐CTO angioplasty. To address this issue, the EURO‐CTO randomized trial [13] included patients only after PCI of non‐CTO lesions, ensuring that any differences in symptoms were attributable solely to CTO treatment.

However, an improvement in NYHA class was observed only in the successful CTO‐PCI group, leading to an increased quality of life for these patients.

### 4.6 Myocardial Ischemia

In this study, a reduction in myocardial ischemia was more frequently observed in the successful CTO‐PCI group compared to the failed CTO‐PCI group and was associated with an improvement in LVEF.

These results are consistent with what is observed in a substudy of the COURAGE trial [39], where PCI in stable CAD resulted in greater ischemia reduction compared to medical therapy.

## 5 Limitations

Our study had several limitations.

First, the observational design precludes causal inference and only allows identification of associations. It may be subject to selection bias and confounding factors. We attempted to minimize this bias through multivariate analyses in which the association remained significant. Although the primary endpoint was adequately powered, subgroup and secondary analyses should be interpreted cautiously due to the smaller number of events in the failed group.

Second, the causes of death were unavailable in half of the cases in the successful CTO‐PCI group and one‐third in the failed group, potentially leading to an underestimation of actual cardiac deaths and MACE rates in both groups.

Third, this is a single‐center study, meaning CTO management practices might differ in other centers. Although the anterograde approach was deliberately chosen to improve patients' outcomes, only 8% of the procedures in our study used the retrograde approach, which might underestimate complication rates, including cardiac tamponade, compared to other practices.

Fourth, we used CCS and NYHA classifications to assess symptoms, which only capture a portion of the patients' quality of life. A more comprehensive and validated tool, such as the Seattle Angina Questionnaire, would provide a better assessment.

Finally, although all patients had OMT, this study did not compare CTO‐PCI with other management options such as CABG and medical therapy alone. Dedicated prospective and randomized studies are therefore needed to determine the clinical benefits of CTO‐PCI.

## 6 Conclusion

This study shows a 15.8% incidence of MACE over a mean follow‐up of 3.7 years in a large cohort of consecutive patients referred for PCI due to CTO at our center. In this population, failed CTO‐PCI was associated with a twofold increase in MACE and rehospitalization rates, without improvements in NYHA class, LVEF, or myocardial ischemia, whereas successful CTO‐PCI achieved improvements in these outcomes.

Further randomized trials are needed to provide clear evidence of the clinical benefits of PCI compared to OMT, enabling the development of reliable recommendations for managing CTOs.

## Author Contributions

**Rémi Arnold:** conceptualization, methodology, formal analysis, investigation, writing – original draft, visualization. **Richard Gervasoni:** conceptualization, investigation, writing – original draft. **Bertrand Ledermann:** investigation. **Lionel Moulis:** methodology, validation, formal analysis, writing – original draft, visualization. **Sonia Soltani:** investigation. **Benoit Lattuca:** investigation. **Pierre Robert:** investigation. **Florence Leclercq:** conceptualization, methodology, investigation, writing – original draft, visualization, supervision, project administration.

## Ethics Statement

Ethical approval for this study was obtained from an independent ethics committee (CSE, Comité Scientifique et Ethique du CHU de Montpellier, number 2024‐01‐008). The study was registered on clinicaltrials.gov (NCT06544174).

## Conflicts of Interest

B.L. has received research grants from Biotronik, Boston Scientific, Daiichi‐Sankyo, Fédération Française de Cardiologie, and Institute of CardioMetabolism and Nutrition; consultant fees from Daiichi‐Sankyo and Eli Lilly; and lecture fees from AstraZeneca, Medtronic, and Novartis. F.L. has received research grants from Boehringer Ingelheim, Edwards Lifesciences, and Medtronic and consultant fees from Bayer AG, Boehringer Ingelheim, and Edwards Lifesciences. P.R. has received research grants from Edwards Lifesciences. The other authors declare no conflicts of interest.

## Data Availability

The data that support the findings of this study are available on request from the corresponding author. The data are not publicly available due to privacy or ethical restrictions.
